# Lipid Emulsion for Local Anesthetic Systemic Toxicity

**DOI:** 10.1155/2012/131784

**Published:** 2011-09-29

**Authors:** Sarah Ciechanowicz, Vinod Patil

**Affiliations:** Department of Anaesthesia, BHR University Hospitals NHS Trust, Romford, London RM7 0AG, UK

## Abstract

The accidental overdose of local anesthetics may prove fatal. The commonly used amide local anesthetics have varying adverse effects on the myocardium, and beyond a certain dose all are capable of causing death. Local anesthetics are the most frequently used drugs amongst anesthetists and although uncommon, local anaesthetic systemic toxicity accounts for a high proportion of mortality, with local anaesthetic-induced cardiac arrest particularly resistant to standard resuscitation methods. Over the last decade, there has been convincing evidence of intravenous lipid emulsions as a rescue in local anesthetic-cardiotoxicity, and anesthetic organisations, over the globe have developed guidelines on the use of this drug. Despite this, awareness amongst practitioners appears to be lacking. All who use local anesthetics in their practice should have an appreciation of patients at high risk of toxicity, early symptoms and signs of toxicity, preventative measures when using local anesthetics, and the initial management of systemic toxicity with intravenous lipid emulsion. In this paper we intend to discuss the pharmacology and pathophysiology of local anesthetics and toxicity, and the rationale for lipid emulsion therapy.

## 1. Introduction

Local anesthetics (LAs) can be defined as drugs that reversibly block transmission of a nerve impulse, without affecting consciousness. Medical use of local anesthetic agents began some years after the isolation of cocaine from Peruvian coca in the 1860s. Chance discovery in 1884 by Freud while using cocaine to wean a morphine addict lead Koller to use cocaine successfully in ophthalmic surgery as a topical anesthetic. Halsted and Hall took more invasive steps by directly injecting cocaine into oral cavity nerves in order to produce anesthesia for removal of a wisdom tooth [1]. 

However, the euphoria, subsequent addiction, and cases of mortality from the clinical use of the natural ester cocaine created a drive to the development of the less toxic newer amino esters. Einhorn's synthesis of procaine in 1905 was to dominate LA use for the next forty years, but with amino esters slow onset of action and allergen potential, the hypoallergenic amino amides gradually came into force with lignocaine appearing in 1948 and is still the most commonly used LA in dentistry. 

Amino amides mepivacaine, prilocaine, and bupivacaine were all developed by 1963 and all have roles in modern dentistry. In 1969, articaine was synthesized by chemist Muschaweck, and with its potency and safety profile is now the most common LA for dental procedures in most of Europe [2]. 

Despite these efforts, all of the amide LAs harbor varying levels of cardiovascular (CVS) and central nervous system (CNS) toxicity that is still a major complication seen today. Methods of administration have also progressed since August Bier first practiced intravenous regional anesthesia in 1908, allowing a whole limb to be anesthetized with the aid of a tourniquet and LA [3]. 

Simultaneously, plexus anesthesia came about in the early 1900s with brachial plexus blocks for upper limb surgeries, these peripheral techniques more refined in recent decades to prolong blocks via continuous infusion regional anesthesia using catheters and pumps [4]. 

The use of LA in neuraxial anesthesia is another significant development that began with James Corning's experiment in 1885 of spinal anesthesia on a dog [5], but it was not used clinically until 1899 by August Bier [6]. Lumbar epidural anesthesia came about later in 1921 by Spanish military surgeon Fidel Pages. It was popularized by the Italian surgeon Dogliotti in the 1930s [7]. 

The idea of continuous infusion of epidural anesthesia, however, was not started until use of caudal blocks for emergency caesareans in 1942 [8], and in more recent decades the introduction of small flexible catheters has improved safety, delivery, and duration of epidural anesthesia.

## 2. Mechanism of Action

**Figure 1:**
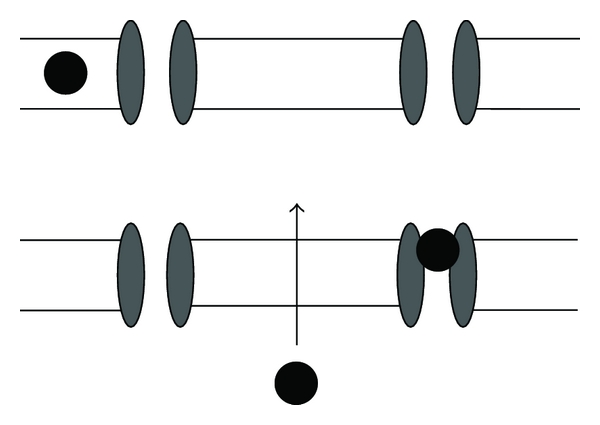
Mechanism of action of local anesthetics. Unionized LA enters nerve axon and becomes ionized to block sodium channels. LA also has direct effects by expanding the cell membrane to increase fluidity.

The physicochemical properties of LAs determine their properties as anesthetic agents. They have three structural groups, an aromatic ring, connecting group (ester or amide), and an ionizable amino group. This lipid-soluble hydrophobic aromatic group and a charged, hydrophilic amide group enables them to exert their effects by two mechanisms: in their uncharged (unionized) state they lipid soluble and able to traverse the lipid bilayer of the neuronal cell membrane, to then gain a hydrogen ion and become ionized making them able to bind intracellularly to voltage-gated sodium channels, rendering the channel reversibly inactive, and so unable to allow for sodium entry to generate and propagate the action potential [9] (see Figure 1). Binding can also occur to the closed sodium channel to retain its inactive state. Secondly, LAs have direct effects on the lipid bilayer, disrupting impulses by incorporating into the cell membrane, causing expansion [10, 11]. The sensitivity of nerve fibers depends upon their axonal diameter and degree of myelination with small, myelinated fibers more susceptible. Generally the small pain and temperature fibers (C unmyelinated, A-*δ* myelinated) are blocked first with the larger touch and pressure (A-*Υ*, A-*β*) fibres next, and large muscle tone and postural A-*α* fibres last. It is thought that the prolonged action potential of smaller fibres provides more time for LA entry, and more frequently stimulated nerves show increased susceptibility from a high degree of open channels. The story does not end there, however, in addition to blocking sodium channels, newer amino amide ropivacaine has been found to bind to human cardiac potassium channels (hKv 1.5) to block repolarization of the membrane [12]. A number of anesthetics, including bupivacaine and ropivacaine, have also been shown to block L-type Ca^2+^ channels in rat cerebrocortical membranes. From a systemic viewpoint, LAs may improve pain by inhibiting local inflammatory response to injury by decreasing inflammatory cytokine release from neutrophils.

## 3. Clinical Pharmacology

Potency is decided by the lipid solubility of the agent and can be expressed as a lipid : water partition coefficient (LWPC), the ratio of the amount of agent in each phase. High coefficients increase lipophilic properties and allows for ease of passage into the cell membrane thus facilitating potency. Onset of action is determined by the ionization constant or pKa value, which determines the proportion of ionized to unionized form of the agent at a given pH. Agents with a pKa value closer to the physiological pH permit more LA in the unionized, lipid soluble form to enter the cell. So factors that alter tissue pH also affect the proportion of LA in the unionized form and hence can slow onset of action in an acidic, infected wound.  Table 1 demonstrates these properties in some common anesthetic agents.

## 4. Pharmacokinetics and Metabolism 

The primary aim of local anesthetic administration is to saturate the targeted nerves while causing minimal systemic absorption. Infiltration of skin, subcutaneous tissues, intrathecal, and epidural spaces will result in varying absorption into the systemic circulation depending on the surface area for absorption and vascularity of the area. Intercostal muscles and epidural administration being particularly susceptible, and in dentistry the gingiva of the maxillary alveolar ridge is prone to inducing rapid systemic absorption. Lignocaine has a vasodilatatory effect and so is often mixed with adrenaline or phenylephrine to reduce vascular absorption and hence prolong action and reduce the risk of systemic toxicity. Conversely, cocaine is a potent vasoconstrictor. 

High protein binding of the LA to plasma protein alpha 1-glycoprotein will protect it from metabolism and hence prolong its duration of action. All amino esters except for cocaine are rapidly degraded by circulating plasma esterases, and excreted in the urine. The amide prilocaine is also metabolized extrahepatically. All other amides such as lignocaine and bupivacaine are more slowly metabolized by the liver and hence are of higher risk of accumulation. 

## 5. Local Anesthetic Systemic Toxicity

### 5.1. Incidence

Before 1981, epidural use for labor analgesia had reported LA systemic toxicity (LAST) in 100 per 10,000 cases [13].

Improvements in regional techniques and precautions have greatly improved the safety profile over the past 30 years, including the withdrawal of higher concentration 0.75% bupivacaine preparations for obstetrics. Although incidence of bupivacaine cardiotoxicity has declined since 1980 it still poses a potentially fatal risk for patients. Epidemiological reports have been clinically diverse and with different outcome measures used, but overall rate of systemic toxicity has been reported in France to be 0–20 per 10,000 in 2002 and is greatly dependent on the site of peripheral nerve block [14]. A study by Brown in 1995 showed seizures associated with interscalene and supraclavicular brachial plexus blocks to be as high as 79 in 10,000 [15].

For example, dentists administer thousands of local anesthetic injections every day with few adverse events. However, LAST can occur even with the most experienced practitioner. Human error misjudging dose, anatomy, patient factors, or bad luck can contribute to the unintended development of serious systemic complications. Lignocaine is the most common LA used in dentistry and has been reported to cause systemic toxicity [16, 17]. Articaine, even with its excellent safety profile, may cause systemic intoxication if unintentional intravascular injection is performed during a block: it has been reported that the rate of intravenous injection for inferior alveolar nerve block is as high as 15.3% [18], which can occur due to the high vascularization of the oral mucosa.

### 5.2. Clinical Manifestations

The signs of LAST are an extension of pharmacological action. The classic description is of a progressive “biphasic” effect on the CNS and then CVS, two areas highly sensitive to changes in tissue electrophysiology. CNS excitation (agitation, auditory change and metallic taste) progresses to seizures or CNS depression (drowsiness, coma, and respiratory arrest). This is followed by CVS excitation (tachycardia, ventricular arrhythmia, and hypertension) then depression (bradycardia, conduction block, asystole, and cardiac depression) [19].

Of particular importance is the nature of this collapse, with high incidence of LA-cardiac arrest being resistant to standard resuscitative measures. 

However, a recent review of 93 published case reports of LAST found that over 40% of presentations did not fit this classic description [20]. This includes the simultaneous presentation of CNS and CVS signs, and cases with only CVS effects manifest. CVS-only effects were seen in 4 out of 10 cases under general anesthesia or form of sedation, and were more likely to show delayed onset of signs. 

Regarding CNS symptoms, the prodromal features, for example, perioral numbness, dizziness, confusion, obtundation, and dysarthria totaled only 18% of symptom frequency, with seizures seen in 68% of cases and loss of consciousness and agitation also frequent. Half of CVS signs were arrhythmias, with bradycardia/asystole seen in 27%.

They reported that timing is variable, for single injections although most onset of LAST occurred “rapidly,” at 50 seconds or less in half of cases, 25% were delayed by 5 minutes or more. Interestingly, all instances of LAST during continuous infusions were substantially delayed, often by a number of days after initiation. 

### 5.3. Toxic Plasma Levels

Systemic toxicity from local anesthetic overdose occurs due to accidental intravascular injection, absorption from tissue depot, or repeated doses without balanced elimination. The concentration of bupivacaine present in the aqueous portion of plasma is directly related to the myocardial tissue absorption, and hence cardiotoxicity [21]. The degree of toxicity is therefore dependent on plasma levels of LA; with highly aerobic tissues vulnerable to hypoxia being most vulnerable, that is, myocardium, lungs and central nervous system. For regional blocks, the plasma levels of lignocaine are typically 3–5 mcg/mL, with toxic plasma levels seen at 6–10 mcg/mL.

### 5.4. Risk Factors

Intuitively, one would speculate that the plasma levels of a given dose of drug would have strong correlation to the weight or body mass index of the individual. In the case of LAs, this is largely true in children, but in adults we see that the methods of administration, nature of the drug preparation, and the physiological status of the patient have far greater association. A poorly vascular injection site of the block, vasoconstrictor activity of the LA, and concurrent use of adrenaline would slow systemic absorption, hence reducing plasma levels, but physiologically, impairment of hepatic and renal function involved in metabolism and elimination can have a profound effect to maintain plasma levels.

Accidental intravascular injection is the major cause of systemic toxicity, for example, regional anesthesia of the neck (interscalene block, cervical plexus block, and stellate ganglion block) can cause direct intra-arterial injection and cause rapid toxicity from early entry to the cerebral circulation. Epidural anesthesia holds a risk of intravenous injection into the engorged epidural venous plexus of the parturient [22], and the oral mucosa is also highly vascular. Regarding site of injection, rapid absorption occurs via infiltration of highly vascular tissues such as intercostal muscles, the oral mucosa, and the epidural space. High cardiac output states also promote systemic uptake by maintaining the gradient for diffusion.

Choice of agent also has clear implications for toxicity. Longer-acting amide LAs such as bupivacaine improve analgesia after surgery and have use in cutaneous infiltration, regional nerve blocks, epidural anesthesia, and spinal anesthesia. However, bupivacaine is more cardiotoxic than shorter-acting lignocaine, with smaller doses often resulting in cardiotoxic symptoms without prior CNS effects [23]. Addition of vasoconstrictors such as epinephrine can dramatically slow the absorption of LAs from the site of injection, improving their safety and prolonging the anesthesia, which is why higher doses of some agents are possible with a vasoconstrictor additive.

Patient physiological factors also have influence on the LA toxicity threshold. Rosen studied the effect of both lignocaine and bupivacaine in anesthetised sheep and found that acidosis, hypoxia, and hypercarbia potentiated cardiotoxic effect [24]. In this sense the elderly are a prime example of risk of imbalance between absorption and metabolism of LA. 

Reduction of hepatic blood flow by drugs or hypotension will decrease the hepatic clearance of amide LAs, and having reduced cardiac output and poor renal or hepatic function leads to prolonged absorption and drug accumulation, respectively. This has implications with use of the recent continuous infusion anesthesia for postoperative orthopedics and acute pain [4, 25]. In addition, use of postoperative pain pumps in plastic surgery can involve bupivacaine combined with epinephrine, which can extend the halflife of bupivacaine from 3.5 hours to 5–7 hours [26]. 

On top of this, underlying cardiac pathology of ischemic heart disease, conduction blocks, and cardiac failure will additionally render the elderly more vulnerable to toxic CVS effects. The majority of the cases of LAST seen in dentistry occur in children, as due to their small size, dose- to- weight ratio is more difficult to calculate and so overdose is more likely. It is also more likely to progress in adversity because a high number of blocks are done with the child anesthetized. The early signs of paraesthesia and mental state changes would not be detected [26]. Conversely, some studies show that newborns and children can actually tolerate higher plasma levels of bupivacaine compared to adults [27, 28]. Kiuchi et al. [29] reports that 2-week old rats (equivalent to 3-year old children) exhibit a lethal dose 4 times higher than 16-week-old animals, and that this difference can be seen as less profound cardiac depression. They speculate this to be due to a difference in calcium regulation at the intracellular sarcoplasmic reticulum. However, in clinical practice, Bosenberg et al. [30] have reported the use of 3 mg/kg of ropivacaine in children without observing symptoms of systemic toxicity or plasma levels of ropivacaine in the range of potential risk for systemic toxicity. 

In pregnancy, the higher cardiac output will speed up absorption and with reduced plasma proteins this will increase the free fraction of LA in the plasma. Plasma protein levels can also vary in different pathological states and there is a reduct-ion seen postoperatively, in chronic diseases such as cancer, also old age, smoking increases the unbound free fraction of agent available to bind to cardiac myocytes and cause toxicity. Lerman et al. [31] have shown that alpha-1 glycoprotein plasma levels are low in newborns and toddlers but the clinical significance of this reduction is not clear. 

Drug interactions are an important patient factor to consider when determining risk of cardiotoxicity. Amide local anesthetics are metabolized by the liver and specifically the cytochrome p450 system that has potential for drug interactions by competitive metabolism and up, or down- regulation of the system by chronic exposure to certain drugs. Cimetidine inhibits the cytochrome p450 system and can allow the accumulation of plasma levels of LAs. Drugs altering plasma esterase activity have the potential to decrease hydrolysis of the lesser-used ester LAs. Increased vigilance is also necessary in patients taking digoxin, calcium antagonists, or beta-blockers [32].

There is debate as to whether general anesthesia provides some protection from toxicity, the effect of general anesthesia in sheep caused plasma LA concentrations to increase due to cardiovascular depression, leading to slower efflux from visc-eral to nonvisceral organs; however, less severe CNS effects and cardiovascular arrhythmias occurred in these sheep [33, 34]. The clinical significance of this is not yet established. For a summary of LAST risk factors see Table 2.

### 5.5. Ion Channels and the Lipid Bilayer

As there are such a myriad of ion channels and processes affected by LAs there is a risk of the culpable mechanism of cardiotoxicity being missed [35]. The pathophysiology of LAs are thought to be an extension of their uses, blocking cardiac voltage-gated sodium channels, preventing myocyte depolarization, blocking repolarization via potassium channels, and blocking the sarcoplasmic reticulum voltage-dependent calcium channels to limit the rise of intracellular calcium available for excitation-contraction coupling [35, 36]. Mio et al. describe a loss of sensitivity of rat ventricular muscle myofilaments to calcium a basis for the loss of calcium-activated tension in trabeculae following access of LA. Furthermore, myocyte ATP is reduced, thus limiting the energy available for coupling of actin-myosin cross-bridge cycles [37]. Work on ion channel involvement is extensive but is not necessarily consistent with cardiotoxicity seen from different agents. Studies on biometric membranes support the notion of increasing lipid membrane fluidity to confer potency of agent and cardiotoxicity [11].

 Animal studies and case reports indicate a difference in cardiotoxicity between short-acting agent lignocaine and the longer-acting bupivacaine. For both agents there is dose-dependent cardiac depression but the greater toxicity potential of bupivacaine is disproportionate and does not correlate entirely with potency of inhibition of cardiac sodium channels. This difference could rely on an alternative mechanism of toxicity for bupivacaine, and we see this clinically in case reports of bupivacaine showing a more significant CVS toxicity than CNS, with arrhythmia and cardiac arrest often occurring without seizures. There appears to be a more potent mechanism occurring at the myocardium, in animal studies lignocaine induces dramatic hemodynamic depression while bupivacaine markedly impairs* both* electrophysiologic and haemodynamic variables [38]. To examine more specifically, Reiz and Nath [39] directly injected lignocaine or bupivacaine into the coronary circulation of dogs and found that the difference in depression of contractility was proportional to their relative potencies, 1 : 4. However, the effect on cardiac conduction was 1 : 16 with recovery of the EKG taking longer for bupivacaine at a ratio of 1 : 8, confirming that the major difference in cardiotoxicity between long-acting and shorter-acting agents is their influence on conduction through the cardiac axis. Clarkson and Hondeghem suggest that bupivacaine has this pronounced effect due to the strength of binding to inactive sodium channels [40]. 

### 5.6. Cardiac Mitochondria

In light of work on the mitochondrial pathogenesis of local anesthetic cardiotoxicity and information from studies and a case report [41] of a child with carnitine deficiency, mitochondrial abnormalities also seem to confer increased susceptibility [42]. Bupivacaine-induced myopathies have led to rat and human cell studies to demonstrate structural alterations in muscle, the sarcomere, and calcium homeostasis by LAs. High bupivacaine concentrations caused abnormal mitochondrial autophagy with reduction in mitochondrial content, inhibition of ATP production by action on mitochondrial ATP-synthase, and inhibition of oxidative phosphorylation [43]. In cardiac tissue, in vivo and vitro studies on rat hearts demonstrate bupivacaine and ropivacaine's ability to uncouple oxidative phosphorylation at complex I in the mitochondria [44], and block the enzyme carnitine acylcarnitine transferase used for transporting acylcarnitines across the mitochondrial membrane in fatty acids during aerobic metabolism [45, 46]. Importantly, inhibition of the respiratory chain complexes was prevented by antioxidant treatment and reversed following removal of the anesthetic thereby suggesting an oxidant-mediated feedback mechanism reinforcing the primary inhibitory action of the anesthetic. Recent developments implicate the mitochondrial phospholipid cardiolipin, involved in respiration, to be the major determinant of LA cardiotoxicity, established by means of theoretic and structural biological methods [47].

### 5.7. Vasoactivity

Secondly to these direct effects on the myocardium, a signif-icant cause of hypotension is due to peripheral vasodilatation from direct action on the vasculature. Bupivacaine and levobupivacaine cause vasodilatation at clinical doses, but lower doses appear to cause vasoconstriction [45]. Direct cardiac depression of bupivacaine has been studied in vivo to demonstrate a deleterious double-whammy on the cardiac output via negative inotropic effects and increasing afterload, which appears to be mediated by *α*1 adrenoceptors [48]. Ropivicaine and levobupivacaine are far less toxic in this sense. 

Thirdly, a mechanism of toxicity appears to be inhibition of autonomic reflexes. There is evidence for inhibition of the baroreceptor reflex in rats [49], and Pickering et al. show bupivacaine to be selectively toxic to the brainstem area for control of cardiac sympathetic outflow, the nucleus tractus solitarius, without effecting respiration, leading to hypotension and dysrhythmias [50]. Lida et al. reveal a differing influence of bupivacaine and ropivacaine on dog spinal pial vessel diameter, with ropivacaine causing vasoconstriction and bupivacaine vasodilatation [51]. Laser doppler imaging studies on human skin has revealed nitric oxide (NO) to be responsible for the vasodilatatory effect of local anaesthetics, however, NO does not appear to be involved when the blood vessel is uninnervated such as the in vitro umbilical artery [52, 53].

## 6. Lipid Emulsion Therapy

20% lipid infusion is the first safe intravenous lipid emulsion (ILE) used in medicine and has been around since 1962 for its use in parenteral nutrition. The commercial preparation Intralipid 20% is manufactured by Fresenius Kabi, 1 liter consists of 200 g purified soybean oil, 12 g purified egg phospholipids, and 22 g anhydrous glycerol, and it is a source of omega-3 and -6 essential fatty acids with total energy content 8.4 MJ (2,000 kCal). ILEs use in LAST came about from an unexpected finding by Weinberg in 1998. Following a case report of a carnitine-deficient patient showing increased susceptibility to bupivacaine cardiotoxicity, he postulated the impaired fatty acid oxidation was the etiology and in seminal work, preloaded rats with ILE prior to bupivacaine in hope to establish this. The result was quite the opposite, with an increase in the mean lethal dose (LD50) by 50% [54]. He later went further to demonstrate the efficacy of ILE by rescuing dogs from bupivacaine-induced cardiac arrest [55]. ILE therapy for treatment of LAST is now well established, following a crop of over 19 peer-reviewed case reports appearing since Rosenblatt's successful application of ILE to clinical practice in 2006 [56], and supports the use of ILE for bupivacaine, levobupivacaine, and ropivacaine cardiotoxicity [56–61]. This year we also saw a successful case report from seemingly intractable lignocaine-induced cardiac arrest [62].

This evidence strongly supports the use of ILE in the resuscitation of LAST and because of this efficacy, ILE is has been incorporated into safety guidelines for management of LA-induced cardiotoxicity in the UK since 2007 and in the US since 2008 [63, 64]. In 2010, the American Society of Regional Anesthesia and Pain Medicine (ASRA) published its practice advisory on LAST [65], highlighting the importance of airway management and early cardiopulmonary resuscitation with addition of ILE therapy. In 2010 the American Heart Association incorporated lipid emulsion for LAST-cardiac arrest in the special situations section of the ACLS guidelines [66].

### 6.1. Mechanism 

The current agreed hypothesis for ILE's efficacy in treating cardiotoxicty, although not well defined but supported by in vitro studies, is the formation of a “lipid sink”; that is, an expanded intravascular lipid phase that acts to absorb the offending circulating lipophilic toxin, hence reducing the unbound free toxin available to bind to the myocardium. The effect of ILE has been disputed to be no more than a haemodilution effect from the volume administered, especially pronounced in rat models [67]. However, convincing evidence from rat studies by Weinberg show ILE to reduce the aqueous plasma bupivacaine concentration three-times greater than that predicted by haemodilution alone [68], and subsequently ILE therapy has shown clear superiority over adrenaline and/or vasopressin in rats that is directly linked to reduced myocardial tissue content and improved cardiac function [21]. Influences on metabolism also seem to confer the success of ILE; there is evidence of increased washout of bupivacaine in rat hearts in the presence of ILE [69]. ILE could be acting as a direct energy source to the myocardium, countering the deleterious effect of LAs on fatty acid delivery by acting as a lipid provider, the fatty acid substrate necessary to enrich mitochondrial respiration in the heart and hence ATP production, thus improving the cardiac output [70]. A further mechanism advocated is that of action of raised triglyceride on cardiac calcium channels to increase myocardial calcium concentration, hence enhancing cardiac function [71]. In addition to its use in LAST, but beyond the scope of this review, is a discussion about the more recent but no less significant discovery of ILE in treatment of cardiotoxicity from a range of other lipophilic drugs including chlorpromazine, beta-blockers, calcium channel antagonists, and bupropion [61]. 

### 6.2. Regimen

**Figure 2:**
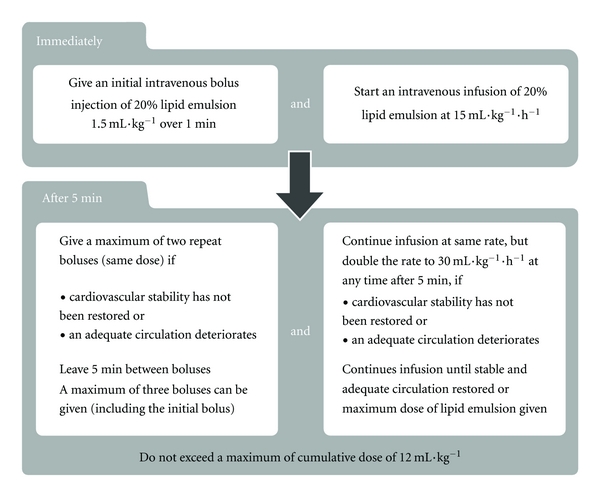
AAGBI local anaesthetic toxicity guideline 2010 (with permission) [63].

The AAGBI recommended ILE or Intralipid regimen following cardiac arrest from LAST involves a large initial intravenous bolus injection of 20% lipid emulsion at 1.5 mL/kg over 1 minute; followed by an infusion of 15 mL/kg/h. Cardiopulmonary resuscitation should be continued throughout. In the absence of return of spontaneous circulation or deterioration after 5 minutes, two further boluses (1.5 mL/kg) may be given at 5-minute intervals. The intravenous infusion rate should also be doubled to 30 mL/kg/hr. A maximum of three boluses can be given, and a cumulative dose of 12 mL/kg should not be exceeded (Figure 2). The ASRA guidelines differ in that only one additional bolus is recommended, and the infusion should continue for 10 minutes after haemodynamic stability is reached, with a maximum dose of 10 mL/kg over 30 minutes [72].

Initial case reports show ILE to often succeed after standard resuscitation has failed and led to the suggestion of ILE as a “last resort” in severe resuscitation resistant LAST. However, there is growing evidence to support its use early in the management with successful case reports supporting the immediate use in cardiac arrest [73–76]. 

Development of optimal dosing regimens for different patient groups in on the horizon, this year ILE has been recommended for use in obstetrics [77]. Support for ILE in pediatric LAST can be seen from a recent case report of ropivacaine and lignocaine-induced toxicity in a 13-year-old girl after lumbar plexus block [57]. Ventricular tachycardia was impressively converted to sinus rhythm after a bolus of 3 mL/kg of lipid emulsion was given over 3 minutes. This is encouraging to read and also poses the question as to whether we need to develop optimal dosing regimens for children. There exists debate about the use of vasopressors with ILE for treatment of LAST, and what combination, if any, is beneficial [78]. Weinberg shows greater survival with ILE alone than with epinephrine and/or vasopressin in rodent models, and combination of ILE and epinephrine worsened outcomes by impairing cardiac function and metabolic indices [79], possibly by worsening coronary perfusion. This is mirrored in the study use of epinephrine and/or vasopressin in cardiac arrest in humans that resulted in early survival but later demise [80, 81]. So perhaps only small doses of epinephrine, if any, are advisable in the treatment of LAST and vasopressin-vasoconstriction is likely to worsen the LA-induced cardiac failure. Further studies are needed to clarify the use of vasopressors in LA-induced cardiac arrest, but at present it is not advised to deviate from standard resuscitation guidelines, with the addition of ILE therapy.

Of interest, the commercial preparation Intralipid may not be the most effective emulsion formulation to use clinically, as described by electrophoresis studies comparing it with liposome vesicle dispersions. The dispersion preparations had increased interaction with local anesthetics compared to standard Intralipid [82], so when financially viable it should be considered for clinical use. There is also discussion of the specific importance of omega-3 fatty acids [83].

## 7. Prevention of Toxicity

Prevention is better than cure, and although no single preventative measure can eliminate the risk of developing LAST, they do provide improved safety. Regarding site of injection, care must be taken to avoid intravascular injection and awareness of tissues prone to rapid uptake, such as the head and neck, is useful. Since the introduction of the measures to prevent inadvertent intravascular injection that began with the epinephrine test dose for labor epidurals by Moore and Batra in 1981 [84], the incidence of LAST has fallen 10–100 fold [85]. The following methods, although singularly unproven, likely promote safety.

- Incremental injection of 3–5 mL aliquots with pause of one circulation time between each, although it increases risk of needle migration. Note circulation time greater in the lower limb.
- Aspirate needle prior to each injection (but 2% false negatives).
- For large volumes, first use intravascular marker, for example, epinephrine 10–15 mcg/mL in adults and 0.5 mcg/kg in children and observe any CVS response.

Although these methods are useful for avoiding intravascular injection, they do not predict the possibility of rapid tissue absorption from the site. To this end, it is important not to exceed the safe dose of local anesthetic involved [86]. The cardiotoxic potential of the amide local anesthetics can be expressed as a maximum safe dose for administration (Table 3). However, for procedures such as tumescent liposuction, the relative avascularity of subcutaneous fat and epinephrine-induced vasoconstriction account for slow lignocaine absorption, and this allows for doses of lignocaine as high as 18 mg/kg to be administered safely.

### 7.1. MLAC and Protocols

The minimum local analgesic concentration (MLAC) of local anesthetics is a clinical model introduced in 1995 to compare the relative potencies of epidural bupivacaine and lignocaine in laboring women. Trials follow up and down sequential allocation of the effective concentration of local anesthetic that produces effective analgesia in 50% of subjects (EC50), to provide an equivalent of the volatile anesthetic “MAC” value [87]. Adoption of this model has allowed for lowest adequate dose regimens and determination of the LA sparing efficacy of adjunct analgesics in obstetrics [88].

### 7.2. US-Guided Regional Anesthesia

Ultrasound (US) can be used to guide the accurate placement of the needle for LA injection over soft tissues, avoiding intravascular injection and damage to surrounding structures and allowing smaller volumes of LA to be used, as direct application to the nerve is more likely. However, systematic review of the Cochrane database finds no difference in the success rate or duration of analgesia between landmark/peripheral nerve stimulator techniques and US-guided blocks, with larger and higher-quality studies lacking [89]. A reduction in incidence of LAST from US has also not yet been proven [90], and there is debate as to whether the reduced volume blocks actually compromise postoperative analgesia [91].

### 7.3. Newer Agents

Stereoisomerism contributes to the differing potency of local anesthetics. Molecules with an asymmetric carbon atom exist in three-dimensional forms that are mirror images (enantiomers and stereoisomers), distinguished by how they rotate polarized light. The terms R and S are used for the two different enantiomers, and an equimolar amount of both R and S constitutes a racemic mixture. Racemic bupivacaine has been in use for decades but is not without its safety concerns. The relatively high toxicity of bupivacaine had led for it to be the main agent implicated in toxicity research. Ropivacaine and levobupivacaine are S-enantiomer pipecoloxylidines that have improved safety profiles compared to racemic bupivacaine. A recent study by Tsuchiya et al. investigating the interaction of racemic bupivacaine and R+ and S-enantiomers of bupivacaine and ropivacaine with biomimetic membranes of chiral lipids demonstrated the greater interaction of the R+ enantiomers, with S-Ropivacaine presenting least influence of all. This is consistent with reported clinical cardiotoxicity of the agents and also supports the hypothesis of potency of increasing the lipid bilayer membrane fluidity [11]. For regional blocks involving sites of high vascularity, the use of alternative long-acting amide levoenantiomers may be vindicated to further reduce the risk to patients, and this has already been suggested in dentistry for interior alveolar nerve blocks [92]. However, a median effective dose study shows ropivacaine and levobupivacaine to, respectively, have 35% and 3% reduced analgesic potency to racemic bupivacaine, and so decisions to use these safer agents must be balanced against a loss of clinical efficacy [93]. 

### 7.4. Surgeon's Awareness

Where LA is provided by nonanesthetists, misdiagnosis and underreporting of LA-associated complications is likely [94]. This includes offices, outpatients, and small surgical centers, and so the true incidence of LAST in these settings is unclear. There are, however, case reports of significant morbidity following LA use in such areas [95–98]. The importance of surgeon's knowledge of safe use of LAs and management of complications is signified by the reported incidence of five deaths from suspected lignocaine systemic toxicity or related complication following tumescent liposuction in New York between 1993–1998 [99–102]. It is of interest to note that this procedure is still very popular today and commonly performed without the presence of an anesthesiologist. Also concerning is a recent survey in the UK by Collins that suggests only half of hospital surgeons know how to calculate the correct dose of local anesthetic being used and fewer than 25% of nonanesthetic doctors knew the recommended safe doses. Only 7% of non-anesthetic doctors knew the correct treatment to be intralipid and only 3% knew the initial dose [103]. These finding highlight the importance of education, which is of particular significance to practitioners who regularly use LAs without the presence of an anesthesiologist.

## 8. Summary

Vigilance is required when performing procedures that have a potential for systemic toxicity. There are numerous examples of local anesthetic systemic complications in the literature, many in the hands of nonanesthesiologists. We see that strategies to reduce the risk of LAST can never eliminate its risk. Although uncommon, the consequences can be fatal. Advances in ILE therapy and understanding is providing a life-saving rescue in the most dreaded situations faced by practitioners, and further progress will likely improve on our safe use of LAs in the future. Rapid identification of toxicity and a good recall of the ILE therapy regimen can save lives, but we need to expand awareness to practitioners in remote locations such as outpatients, offices, and especially those who work without an anesthesiologist. We encourage these facilities to put together a “rescue kit” in a specified location with the current guidelines readily available. LAs are used more frequently by surgeons and dentists than anesthesiologists, and on that note we feel that the respective colleges should also develop guidelines for management of LAST incorporating lipid emulsion therapy.

## Figures and Tables

**Table 1:** Pharmacology of common local anesthetics. Potency is relative. Potency: toxicity ratio is a useful evaluation to consider, articaine has the best ratio making it clinically efficacious as well as safe. %PB = protein binding.

	Potency	Pot : Tox	LWPC	Onset	pKa	*t* ^1/2^ ** (**min**)**	%PB
Bupivacaine	8	2	27.5	Slow	8.1	162	95.6
Articaine	3	3.3	17	Fast	7.8	20	94
Lignocaine	2	2	2.9	Fast	7.9	96	64.3
Mepivacaine	2	2.2	19.3	Fast	7.8	114	78
Prilocaine	2	2.7	0.9	Fast	7.7	93	55
Ropivacaine	4	2.25	2.9	Mod	8.1	96	94

**Table 2:** Factors affecting LA toxicity.

Site of injection	Drug	Patient factors
Surface area Vascularity	Potency Dose *(volume *×* concentration) * Vasoactivity ± vasoconstrictor	Age Genetics Cardiac pathology Pregnancy Drug interactions Acidosis Hypoxia Hypercarbia

**Table 3:** Safe doses of common LAs.

	Maximum safe dose (mg/kg)
Bupivacaine	2.0
Levobupivacaine	2.5–3.0
Articaine	7.0
Lignocaine	4.0
*with epinephrine*	7.0
Mepivacaine	7.0
Prilocaine	6.0
Ropivacaine	3.0-4.0
